# *SPP1* and *CXCL9* Promote Non-alcoholic Steatohepatitis Progression Based on Bioinformatics Analysis and Experimental Studies

**DOI:** 10.3389/fmed.2022.862278

**Published:** 2022-04-19

**Authors:** Wen Wang, Xiaojing Liu, Peiyao Wei, Feng Ye, Yunru Chen, Lei Shi, Xi Zhang, Jianzhou Li, Shumei Lin, Xueliang Yang

**Affiliations:** ^1^Department of Infectious Diseases, The First Affiliated Hospital of Xi'an Jiaotong University, Xi'an, China; ^2^Department of Nutrition, The First Affiliated Hospital of Xi'an Jiaotong University, Xi'an, China

**Keywords:** NASH, *SPP1*, *CXCL9*, bioinformatics analysis, MCD diet

## Abstract

**Background and Aims:**

Non-alcoholic fatty liver disease (NAFLD) is a major chronic liver disease worldwide, and non-alcoholic steatohepatitis (NASH) is one of its pathological subtypes. The pathogenesis of NASH has not yet been fully elucidated. The purpose of this study was to identify the hub genes and pathways involved in NASH using bioinformatics methods. The hub genes were confirmed in human and animal models.

**Materials and Methods:**

Three Gene Expression Omnibus (GEO) datasets (GSE48452, GSE58979, and GSE151158) of NASH patients and healthy controls were included in the study. We used GEO2R to identify differentially expressed genes (DEGs) between NASH patients and healthy controls. Functional enrichment analyses were then performed to explore the potential functions and pathways of the DEGs. In all DEGs, only two genes were highly expressed in NASH patients throughout the three datasets; these two genes, *SPP1* and *CXCL9*, were further studied. Serum and liver tissues from NASH patients and healthy controls were collected. Serum alanine aminotransferase (ALT) and aspartate aminotransferase (AST) levels were measured in NASH patients and healthy controls. Liver tissues were stained with hematoxylin and eosin. Immunohistochemical staining was used to evaluate the expression levels of the two genes in liver tissues. Male C57BL/6J mice were fed a methionine choline-deficient (MCD) diet for 8 weeks, after which serum ALT and AST levels were measured and liver tissues were stained.

**Results:**

*SPP1* and *CXCL9* were the hub genes detected in the three datasets. “Lipid metabolism,” “inflammatory response,” and “lymphocyte activation” were the most significant biological functions in GSE48452, GSE58979, and GSE151158, respectively. Kyoto Encyclopedia of Genes and Genomes pathway analysis showed that the toll-like receptor signaling pathway was significantly enriched in NASH patients. Serum ALT and AST levels were significantly increased in NASH patients compared to healthy controls. Liver tissues had more serious steatosis, hepatocyte ballooning degeneration, and lobular inflammatory infiltration, and the expression of *SPP1* and *CXCL9* in liver cells was significantly upregulated in NASH patients compared to healthy controls. MCD diet mice were consistent with NASH patients.

**Conclusion:**

*SPP1* and *CXCL9* may play important roles in NASH pathogenesis and could be potential therapeutic targets and biomarkers of NASH in the future. Further experimental studies are needed to confirm our results.

## Introduction

Non-alcoholic fatty liver disease (NAFLD) is pathologically classified as non-alcoholic fatty liver (NAFL) or non-alcoholic steatohepatitis (NASH) based on the presence of ballooned hepatocytes (1). NASH is characterized by steatosis, ballooning necrosis around steatotic hepatocytes, mild inflammation, and fibrosis (2, 3). The global prevalence of NAFLD in the general population is estimated to be 25%, whereas that of NASH is estimated to range from 3 to 5% (4). Over the past 20 years, the prevalence of NAFLD in China has doubled, and the disease is most prevalent in the urban population (27%). Globally, China is expected to have the fastest increase in NAFLD incidence due to urbanization (5). As the prevalence of NAFLD continues to rise, NASH has the potential to become the most common cause of cirrhosis and end-stage liver disease in the coming decades (6).

Currently, the pathogenesis of NASH is unclear. The “two hit hypothesis” is a classic theory that describes the development and progression of NAFLD/NASH, but this view is too simplistic to recapitulate the molecular and metabolic changes that underlie the pathogenesis of NASH (7, 8). Recently, a “multiple-hit hypothesis” has replaced the “two hit hypothesis” to explain disease pathogenesis in NASH (9). This theory suggests that multiple factors, such as insulin resistance, hormones secreted by adipose tissue, nutrition, lifestyle, gut microbiota, inflammatory genes, and genetic and epigenetic factors, work together to induce NASH in genetically predisposed subjects, providing a more accurate explanation for the pathogenesis of NASH (10–14). At present, the management of NASH primarily focuses on diet and exercise, but still fails to achieve good clinical outcomes. NASH pharmacotherapy is under study, and to date, there are no Food and Drug Administration-approved drugs to treat NASH (15). It is important to understand the precise molecular mechanisms of NASH pathogenesis and develop and identify therapeutic targets for effective diagnosis, management, and therapy. Thus, determining key genes that affect the occurrence and development of NASH can contribute to the elucidation of its molecular mechanisms and the identification of new diagnostic markers and therapeutic targets.

Therefore, we analyzed differentially expressed genes (DEGs) in the liver tissues of patients with NASH using data downloaded from the Gene Expression Omnibus (GEO) database to identify the hub genes involved in the pathogenesis of the disease. Next, the potential mechanisms of the DEGs in NASH were explored using Gene Ontology (GO) and Kyoto Encyclopedia of Genes and Genomes (KEGG) pathway analyses. Finally, we verified the hub genes in the liver tissues of NASH patients and methionine choline-deficient (MCD) diet mice; these hub genes may be candidate diagnostic or therapeutic biomarkers for NASH.

## Materials and Methods

### Microarray Data Collection

We obtained the gene expression datasets of NASH from the Gene Expression Omnibus (GEO) database (http://www.ncbi.nlm.nih.gov/geo/). We systematically searched the studies using the following terms: “fat liver,” “non-alcoholic fatty liver disease,” “non-alcoholic steatohepatitis,” and “*Homo sapiens*.” The gene expression profiles of GSE48452, GSE58979, and GSE151158 were downloaded. The NASH and healthy control (HC) patient sample sizes were 18 vs. 14 in GSE48452, 8 vs. 10 in GSE58979, and 17 vs. 21 in GSE151158, respectively.

### Identification of DEGs

The original microarray data were analyzed using the online tool GEO2R (www.ncbi.nlm.nih.gov/geo/geo2r), which allows researchers to identify DEGs in each group. Genes with *P* < 0.05 and |log FC| > 1.5 were considered as DEGs. The overlapping DEGs in all three datasets were analyzed using Venn diagrams (http://bioinformatics.psb.ugent.be/webtools/Venn/).

### Function Enrichment Analyses

GO provides comprehensive information on the gene function of individual genomic products based on defined features comprising molecular function, biological processes, and cellular components. KEGG is a biological information database for understanding high-level biological functions and utilities. These analyses and annotations were performed using the Database for Annotation, Visualization, and Integrated Discovery (https://david.ncifcrf.gov/), which provides functional annotation information regarding genes and proteins for researchers to explore the biological meaning of a specified gene list. Statistical significance was set at *P* < 0.05.

### Human Samples

NASH liver tissues and blood samples were obtained from morbidly obese patients undergoing bariatric surgery, and normal liver tissues and blood samples were taken from adults without obesity who underwent liver resection for hemangioma. The study was conducted according to the principles of the Declaration of Helsinki, and written informed consent was obtained from all patients. The study was reviewed and approved by the Ethics Committee of the First Affiliated Hospital of Xi'an Jiaotong University (ethics approval number: 2018QN-13).

### Animal Experiments

Male C57BL/6J mice (6-week-old) were supplied by the Experimental Animal Center of Xi'an Jiaotong University. Mice were housed in an animal center with a light-dark cycle of 12 h and free access to food and water. They were randomly divided into two groups: standard chow diet (HC group, *n* = 5) and MCD diet (NASH group, *n* = 5). After 8 weeks of feeding, liver tissues and blood samples were collected for the subsequent experiments. All experimental procedures were approved by the Animal Ethics Committee of Xi'an Jiaotong University (ethics approval number: 2018QN-13) and followed the animal ethical guidelines.

### Hematoxylin and Eosin (H&E) Staining and Immunohistochemistry

Liver tissues were fixed, dehydrated, cleared, and immersed in paraffin wax. The paraffin-embedded tissues were sliced into 3–4 μm-thick sections. All samples from NASH patients, HCs, and mice were reviewed histologically using H&E staining. Immunohistochemistry was performed using an anti-SPP1 antibody (Abclonal, Wuhan, China) and anti-CXCL9 antibody (ThermoFisher, Waltham, MA, USA).

### Serum Analysis

Serum alanine aminotransferase (ALT) and aspartate aminotransferase (AST) levels were evaluated to assess liver inflammation using an LST008 Biochemistry Analyzer (Hitachi, Ltd., Tokyo, Japan).

### Statistical Analysis

All data are presented as the mean ± standard deviation. Statistical analysis between groups used an unpaired two-tailed Student's *t*-test. Statistical significance was set at *P* < 0.05. All analyses were performed in GraphPad Prism 6 (GraphPad Software, San Diego, America).

## Results

### Identification of DEGs in NASH

**Figure 1:**
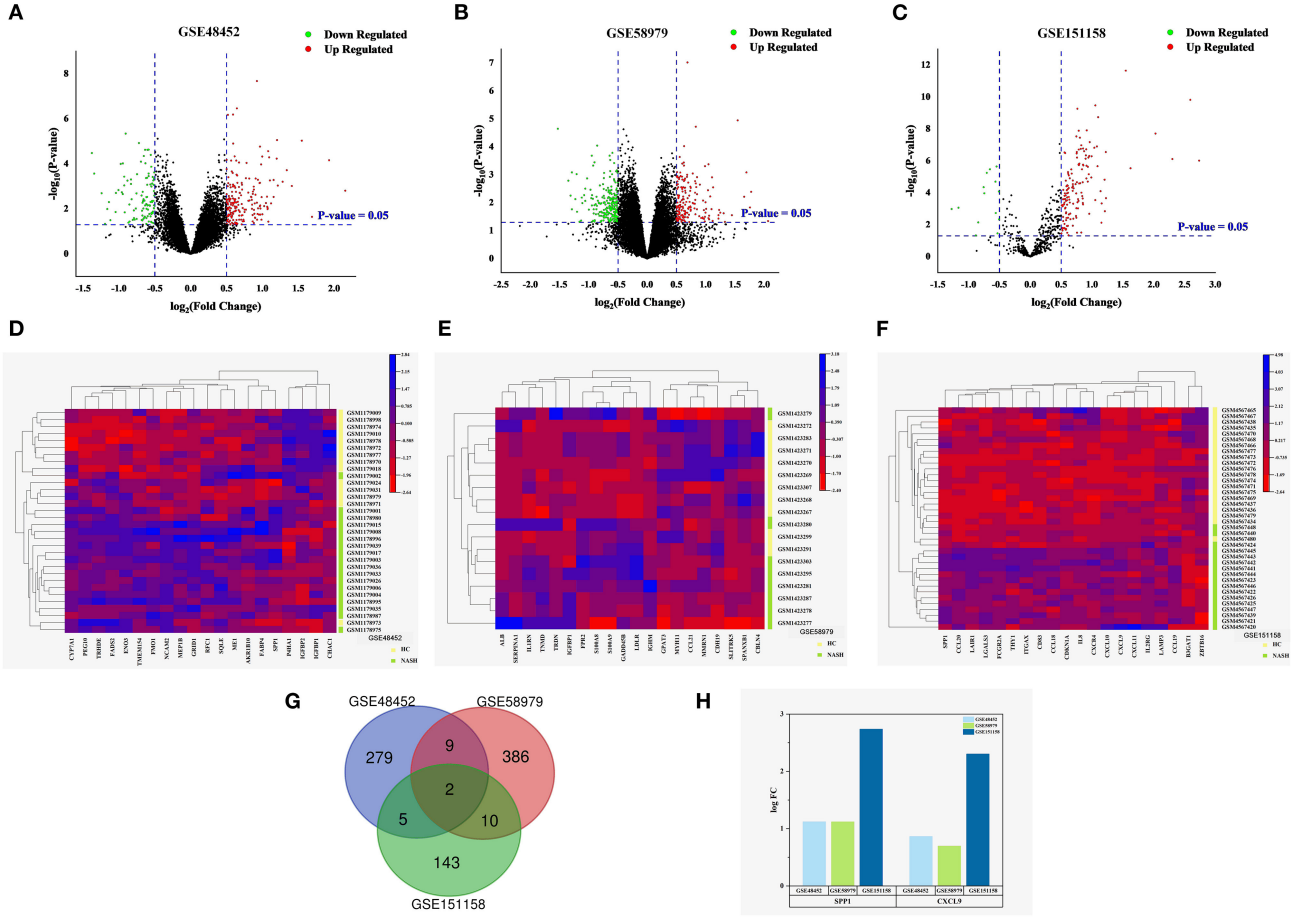
Identification of DEGs in NASH. **(A–C)** Volcano plot of the differentially expressed genes of the three datasets. Red points indicate DEGs with a logFC > 0.5 and *P* < 0.05, green points indicate DEGs with a logFC < −0.5 and *P* < 0.05. **(A)** GSE48452. **(B)** GSE58979. **(C)** GSE151158. **(D–F)** Heat map for the top 20 DEGs between NASH and normal liver tissues of the three datasets. Green lines represent NASH, and yellow lines represent HC. **(D)** GSE48452 (containing 18 NASH tissues and 14 normal liver tissues). **(E)** GSE58979 (8 NASH tissues and 10 normal liver tissues). **(F)** GSE151158 (17 NASH tissues and 21 normal liver tissues). **(G,H)** Venn diagram and the expression levels of the intersecting genes in GSE48452, GSE58979, and GSE151158. **(G)** Venn diagram displaying two DEGs present in both NASH and HC. **(H)** Expression levels of the intersecting genes (logFC = log Fold Change).

Three datasets were downloaded from the GEO database: GSE48452, GSE58979, and GSE151158. A total of 295 DEGs were identified in the GSE48452 dataset, including 194 upregulated and 101 downregulated genes (Figure 1A). Additionally, 407 DEGs were identified in the GSE58979 dataset, including 178 upregulated genes and 229 downregulated genes (Figure 1B). Finally, 160 DEGs were identified in the GSE151158 dataset, including 146 upregulated genes and 14 downregulated genes (Figure 1C). Heatmaps indicated the expression patterns of the top 20 DEGs in the datasets GSE48452 (*CYP7A1, PEG10, TRHDE, FADS2, ENO3, TMEM154, FMO1, NCAM2, MEP1B, GRID1, RFC1, SQLE, ME1, AKR1B10, FABP4, SPP1, P4HA1, IGFBP2, IGFBP1, CHAC1*), GSE58979 (*ALB, SERPINA1, IL1RN, TNMD, TRDN, IGFBP1, FPR2, S100A8, S100A9, GADD45B, LDLR, IGHM, GPAT3, MYH11, CCL21, MMRN1, CDH19, SLITRK5, SPANXB1, CBLN4*), and GSE151158 (*SPP1, CCL20, LAIR1, LGALS3, FCGR2A, THY1, ITGAX, CD83, CCL18, CDKN1A, IL8, CXCR4, CXCL10, CXCL9, CXCL11, IL2RG, LAMP3, CCL19, B3GAT1, ZBTB16*) (Figures 1D–F). To compare the NASH and HC groups, the intersection of DEGs from these datasets was presented in a Venn diagram and two upregulated genes were subsequently identified: *SPP1* and *CXCL9* (Figures 1G,H).

### Functional Enrichment Analysis of DEGs

**Figure 2:**
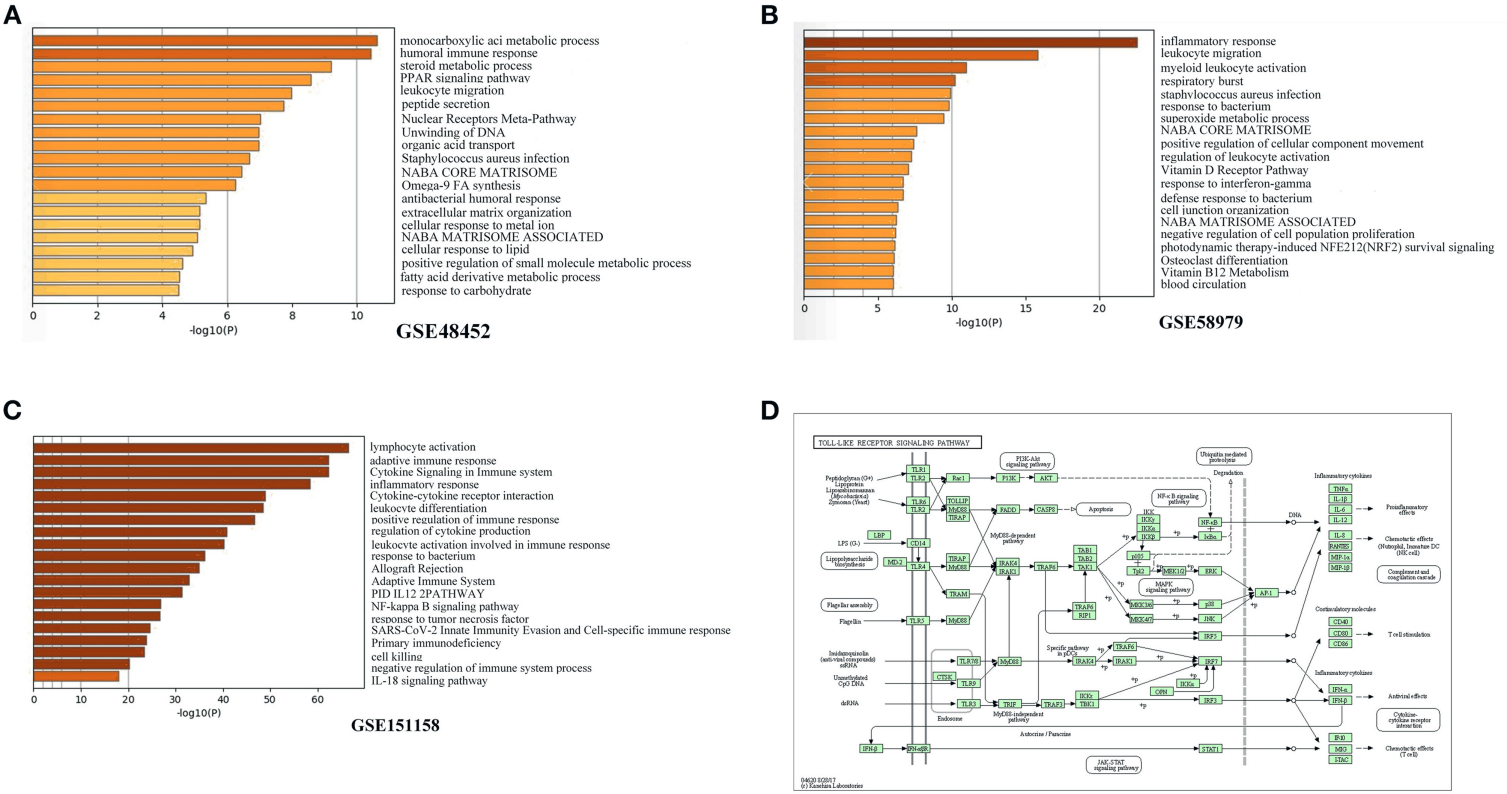
Functional enrichment analysis of DEGs. **(A–C)** Top 20 biological functions according to gene ontology (GO) analysis of the two DEGs. **(A)** GSE48452. **(B)** GSE58979. **(C)** GSE151158. **(D)** KEGG functional analysis: colored map of the toll-like receptor signaling pathway.

GO analysis was applied to investigate the biological functions of the identified DEGs in the three datasets. The biological process analysis in GSE48452 primarily included lipid metabolism (e.g., steroid metabolic process, peroxisome proliferator-activated receptor signaling pathway, cellular response to lipids, and fatty acid derivative metabolic process) (Figure 2A). The biological process analysis in GSE58979 mainly included inflammatory response (e.g., leukocyte migration, myeloid leukocyte activation, regulation of leukocyte activation, and response to interferon-gamma) (Figure 2B). Similarly, the biological process analysis in GSE151158 primarily included inflammatory response (e.g., lymphocyte activation, adaptive immune response, cytokine signaling in the immune system, cytokine-cytokine receptor interaction, leukocyte differentiation, and positive regulation of immune response) (Figure 2C).

KEGG pathway analysis was utilized to elucidate the pathway based on the two DEGs identified previously. The toll-like receptor signaling pathway was significantly enriched in KEGG pathways in NASH patients (hsa04620; *P* = 0.015) (Figure 2D).

### *SPP1* and *CXCL9* Were Significantly Upregulated in NASH Patients

**Figure 3:**
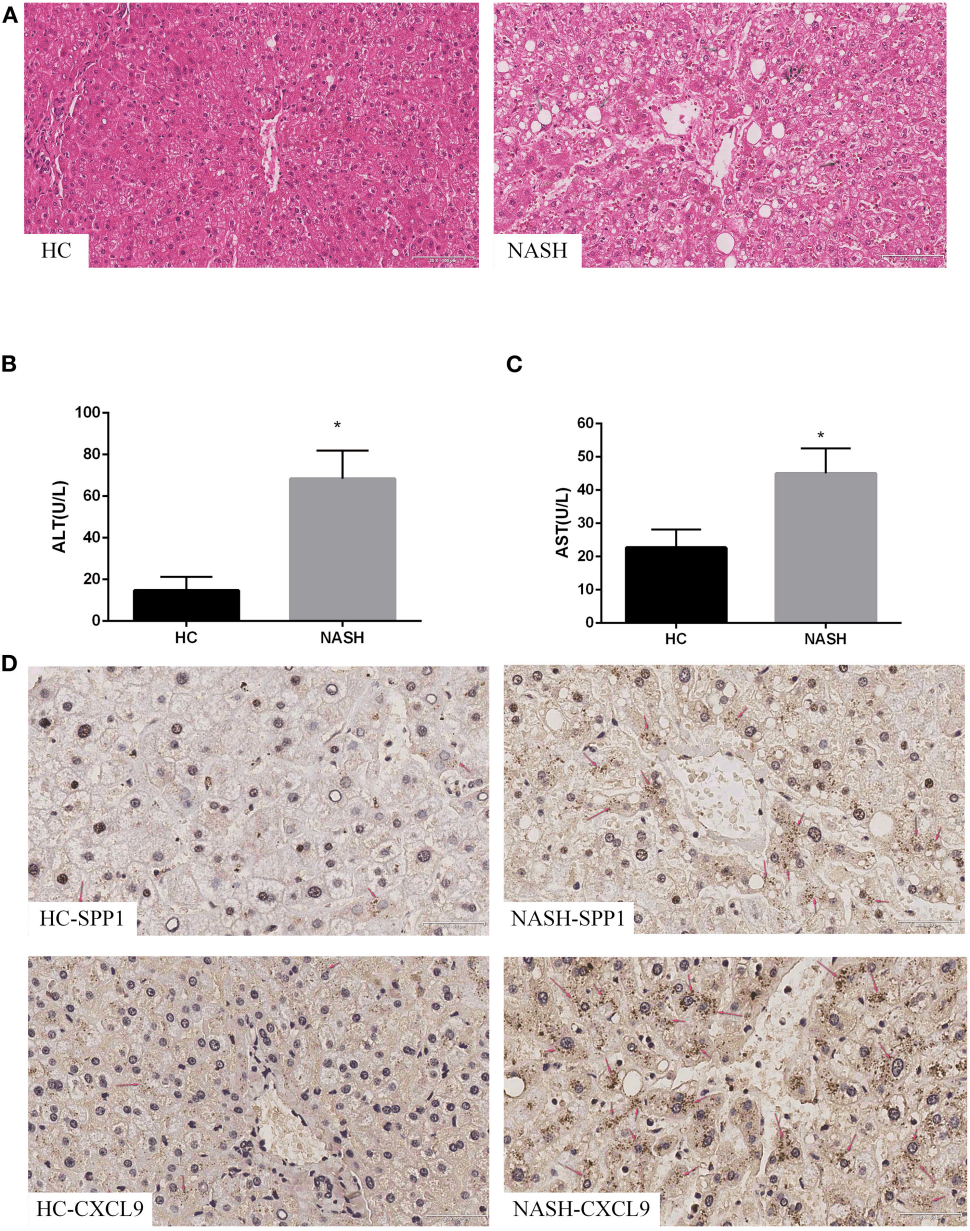
Expression of *SPP1* and *CXCL9* in NASH patients. Serum biochemical indices change, morphological analysis and immunohistochemistry images of the expression of *SPP1* and *CXCL9* in NASH and HC group of human liver samples. **(A)** The liver samples were stained with hematoxylin–eosin (20×) in NASH and HC groups. The gray arrows represent steatosis, the red arrows represent hepatocyte ballooning degeneration, and the black arrows represent lobular infiltration. **(B,C)** Serum content of ALT and AST changes between HC patients and NASH patients. ALT and AST concentrations were significantly higher in NASH group than those in HC group. *n* = 5, *P < 0.05 compared with HC group. **(D)** Determination of the expression levels of *SPP1* and *CXCL9* in NASH and HC group of human liver samples using immunohistochemical staining (40×). The red arrows represent *SPP1* or *CXCL9* proteins in liver cells.

To further validate *SPP1* and *CXCL9*, identified using bioinformatics analysis, we examined the expression of the two DEGs in NASH patients and HCs. H&E staining confirmed that NASH patients had more serious steatosis, hepatocyte ballooning degeneration, and lobular inflammatory infiltration than the HC group (Figure 3A). Serum ALT and AST levels were significantly elevated in NASH patients compared to the HC group (Figures 3B,C). Immunohistochemical results showed that the expression levels of *SPP1* and *CXCL9* were significantly upregulated in NASH liver tissues compared to healthy liver tissues, which is consistent with the results of the bioinformatics analysis mentioned previously (Figure 3D).

### *SPP1* and *CXCL9* Were Significantly Upregulated in MCD Diet Mice

**Figure 4:**
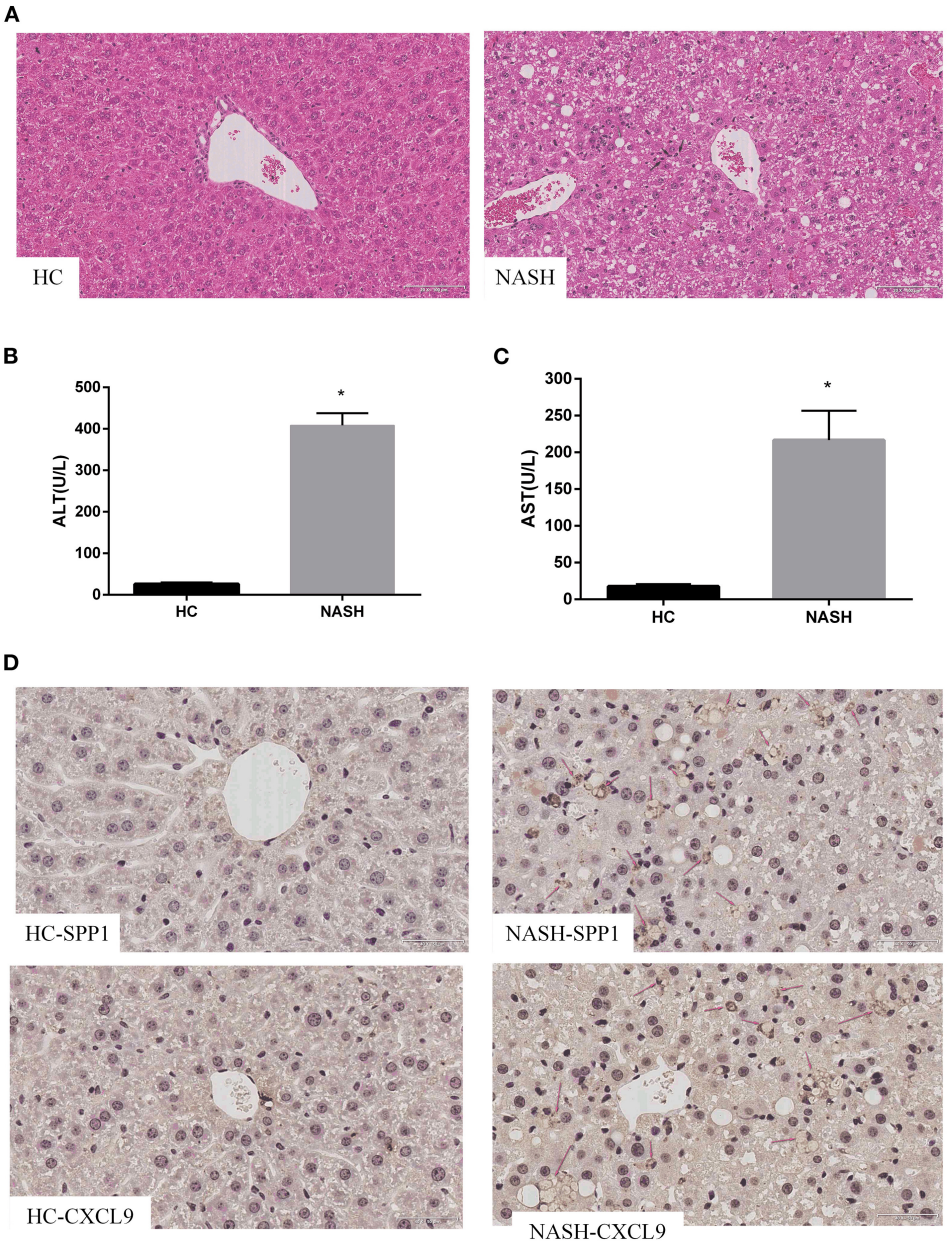
Expression of *SPP1* and *CXCL9* in MCD diet mice. Serum biochemical indices change, morphological analysis and immunohistochemistry images of the expression of *SPP1* and *CXCL9* in NASH and HC group of mice liver samples after MCD diet treatment for 8 weeks. **(A)** The liver samples were stained with hematoxylin–eosin (20×) in NASH and HC groups. The gray arrows represent steatosis, the red arrows represent hepatocyte ballooning degeneration, and the black arrows represent lobular infiltration. **(B,C)** Serum content of ALT and AST changes after MCD diet treatment for 8 weeks. ALT and AST concentrations were significantly higher in NASH group than those in HC group. *n* = 3, *P < 0.05 compared with HC group. **(D)** Determination of the expression levels of *SPP1* and *CXCL9* in NASH and HC group of mice liver samples using immunohistochemical staining (40×). The red arrows represent *SPP1* or *CXCL9* proteins in liver cells.

Based on the results in human samples, we further verified the expression of *SPP1* and *CXCL9* in an MCD diet mouse model. H&E staining confirmed that MCD diet mice had more serious steatosis, hepatocyte ballooning degeneration, and lobular inflammatory infiltration than the HC group (Figure 4A). Serum ALT and AST levels in the MCD diet mice were significantly higher than those in the HC group (Figures 4B,C). Immunohistochemical results showed that the expression levels of *SPP1* and *CXCL9* were significantly upregulated in MCD diet mice compared to HC mice, which is consistent with the above results (Figure 4D).

## Discussion

In this study, bioinformatics analysis was utilized to identify DEGs related to NASH. The potential role of DEGs in NASH was explored using GO and KEGG pathway analyses. The hub genes were verified in the liver tissues of NASH patients and HC patients. We identified two hub genes, *SPP1* and *CXCL9*, both of which had upregulated expression levels. Functional enrichment analysis showed that DEGs were mainly enriched in lipid metabolism, inflammatory response, and lymphocyte activation in these datasets. The toll-like receptor signaling pathway was the main enrichment pathway for the hub genes. GO and KEGG pathway analyses indicated that one of the mechanisms of NASH pathogenesis involves changes that affect metabolism and stress responses (16). The upregulation of *SPP1* and *CXCL9* genes in NASH livers was also confirmed in patient and mice samples. These results may contribute to the understanding of the pathogenesis of NASH and identification of potential diagnostic or therapeutic biomarkers.

Secreted phosphoprotein 1 (SPP1), also known as Osteopontin (OPN), is a secreted phosphorylated glycoprotein. *SPP1* is seldomly expressed in normal liver tissue and is mainly expressed in activated Kupffer cells, hepatic macrophages, hepatic stellate cells, and hepatocytes under pathological conditions (17–19). As a multifunctional protein, SPP1 is involved in multiple liver diseases by promoting inflammatory responses, cell activation, proliferation, and migration, and is closely related to the occurrence, development, and prognosis of fatty liver, liver fibrosis, and liver cancer (20, 21). Previous studies have revealed that the expression of *SPP1* is upregulated in NAFLD and NASH liver tissues. In a new swine model of NASH, *SPP1* gene expression was significantly positively correlated with lipid droplet area and inflammation, and when NASH was reversed, *SPP1* gene expression was significantly reduced (22). A cross-sectional survey of 19 NASH patients reported that plasma SPP1 levels in NASH patients were higher than those in HC patients, whereas plasma and hepatic SPP1 levels in patients with advanced fibrosis were higher than those in patients with no or mild fibrosis, indicating that the histological characteristics of disease severity in NASH patients are associated with SPP1 (23). Serum SPP1 levels were measured in 179 patients with NAFLD and 123 HC patients and were significantly elevated in patients with NAFLD, and were independently associated with liver enzymes and portal inflammation (24). Inhibition of SPP1 can improve inflammation in adipose and liver tissues (25). Furthermore, other studies have confirmed that knocking out the *SPP1* gene can aggravate the progression of fatty liver disease. SPP1 deficiency may reduce liver-related mortality, which may be associated with the promotion of a systemic pro-inflammatory milieu by *SPP1*. This suggests that *SPP1* plays a dual role in the pathogenesis of fatty liver and that *SPP1* may be involved in the internal control of excessive lipid uptake in the liver, thereby protecting the liver from lipotoxicity, apoptosis, and subsequent fibrosis and hepatocyte proliferation (26).

The expression of C-X-C motif chemokine ligand 9 (*CXCL9*) was also upregulated in the liver tissues of patients with NASH. The *CXCL9* gene is a member of a chemokine superfamily that encodes secreted proteins involved in immunoregulatory and inflammatory processes and is thought to be involved in T cell trafficking. The encoded protein binds to C-X-C motif chemokine 3 (*CXCL3*) and is a chemoattractant for lymphocytes but not for neutrophils. *CXCL9* has been reported to be involved in various pathological processes, including tumor development, immunity, and inflammation (27, 28). Overexpression of *CXCL9* mRNA was observed in both NASH and simple steatosis mouse models, and hepatocytes and sinusoid endothelial cells secreting CXCL9 protein were localized in areas infiltrated with inflammatory cells (29). The expression of *CXCL9* was upregulated in NASH without fibrosis in a high-risk cohort of adults with obesity (12). Another cohort study showed that the serum concentration of *CXCL9* in patients with chronic liver disease was significantly higher than that in HC patients and was positively correlated with the severity of liver fibrosis (30). Increased expression of *CXCL9* was also found in hepatocytes of patients with chronic hepatitis C virus infection, and its expression levels were associated with liver fibrosis (31). Overexpression of *CXCL9* is associated with tumor progression. In mouse models, a chronically increasing trend in *CXCL9* levels was associated with the progression from NAFLD to hepatocellular carcinoma in male mice (32, 33). These studies suggest that *CXCL9* is an important factor in chronic liver inflammation and that its expression and role in NAFLD require further research.

Atherosclerosis is the leading cause of death in NAFLD and NASH patients. Chemokines play an important role in NAFLD and NASH with atherosclerosis. IL-17, which is released by the visceral adipose tissue, can induce Eotaxin secretion via the smooth muscle cells in atherosclerotic blood vessels. Eotaxin is another member of the CC chemokine subfamily and was able to predict carotid intima-media thickness (IMT), a marker of atherosclerosis (34). CXCL9 expression also may be regulated by IL-17. IL-17 can upregulate the expression of chemokines, such as CXCL9, in mice with hypersensitivity pneumonitis, while IL-17 gene-deficient mice had reduced levels of CXCL9 (35). Obesity and insulin resistance are central to NAFLD and hepatic steatosis. Stem cell growth factor-beta (SCGF-β) levels were positively correlated to insulin resistance and hepatic steatosis severity in a retrospective study on obese patients (36). In addition, CXCL9 and SCGF-β levels were higher in unstable plaques compared to stable plaques (37). The relationship between CXCL9 and SCGF-β in NAFLD requires further research.

In conclusion, using bioinformatics analysis of NASH patients and healthy controls, we identified two hub genes (*SPP1* and *CXCL9*), which were significantly upregulated. The upregulation of *SPP1* and *CXCL9* in NASH livers was also verified using human and mouse samples. Our study confirmed that *SPP1* and *CXCL9*, which play key roles in NASH pathogenesis, are potential targets for the prevention and treatment of NASH. However, the findings of this study need to be validated by further experimental studies.

## Data Availability Statement

The datasets presented in this study can be found in online repositories. The names of the repository/repositories and accession number(s) can be found below: http://www.ncbi.nlm.nih.gov/geo/.

## Ethics Statement

The studies involving human participants were reviewed and approved by the Ethics Committee of The First Affiliated Hospital of Xi'an Jiaotong University. The patients/participants provided their written informed consent to participate in this study. The animal study was reviewed and approved by the Animal Ethics Committee of Xi 'an Jiaotong University.

## Author Contributions

WW and XL contributed to the data analysis, experiments, and manuscript writing. PW, FY, and YC contributed to the data analysis and the data interpretation. LS and XZ conducted the experiments and analyzed the data. JL wrote and revised the manuscript. SL and XY conceived and designed the study and wrote the manuscript. All authors read and approved the submitted version.

## Funding

This study was supported by the National Major Scientific and Technological Project (Nos. 2017ZX10201201 and 2017ZX10202202) and Institutional Foundation of The First Affiliated Hospital of Xi'an Jiaotong University (No. 2018QN-13).

## Conflict of Interest

The authors declare that the research was conducted in the absence of any commercial or financial relationships that could be construed as a potential conflict of interest.

## Publisher's Note

All claims expressed in this article are solely those of the authors and do not necessarily represent those of their affiliated organizations, or those of the publisher, the editors and the reviewers. Any product that may be evaluated in this article, or claim that may be made by its manufacturer, is not guaranteed or endorsed by the publisher.
